# Multiplex Microsphere Immunoassays for the Detection of IgM and IgG to Arboviral Diseases

**DOI:** 10.1371/journal.pone.0075670

**Published:** 2013-09-25

**Authors:** Alison J. Basile, Kalanthe Horiuchi, Amanda J. Panella, Janeen Laven, Olga Kosoy, Robert S. Lanciotti, Neeraja Venkateswaran, Brad J. Biggerstaff

**Affiliations:** 1 Division of Vector-Borne Diseases, Centers for Disease Control, Fort Collins, Colorado, United States of America; 2 Radix Biosolutions, Georgetown, Texas, United States of America; University of Texas Medical Branch, United States of America

## Abstract

Serodiagnosis of arthropod-borne viruses (arboviruses) at the Division of Vector-Borne Diseases, CDC, employs a combination of individual enzyme-linked immunosorbent assays and microsphere immunoassays (MIAs) to test for IgM and IgG, followed by confirmatory plaque-reduction neutralization tests. Based upon the geographic origin of a sample, it may be tested concurrently for multiple arboviruses, which can be a cumbersome task. The advent of multiplexing represents an opportunity to streamline these types of assays; however, because serologic cross-reactivity of the arboviral antigens often confounds results, it is of interest to employ data analysis methods that address this issue. Here, we constructed 13-virus multiplexed IgM and IgG MIAs that included internal and external controls, based upon the Luminex platform. Results from samples tested using these methods were analyzed using 8 different statistical schemes to identify the best way to classify the data. Geographic batteries were also devised to serve as a more practical diagnostic format, and further samples were tested using the abbreviated multiplexes. Comparative error rates for the classification schemes identified a specific boosting method based on logistic regression “Logitboost” as the classification method of choice. When the data from all samples tested were combined into one set, error rates from the multiplex IgM and IgG MIAs were <5% for all geographic batteries. This work represents both the most comprehensive, validated multiplexing method for arboviruses to date, and also the most systematic attempt to determine the most useful classification method for use with these types of serologic tests.

## Introduction

Arthropod-borne viruses (arboviruses) are responsible for considerable morbidity and mortality worldwide. Those most heavily affected live at tropical latitudes where mosquitoes are most active and difficult to control [1]. Human vaccines are available for yellow fever (YF), Japanese encephalitis (JE) and tick-borne encephalitis (TBE) viruses, and long-sought vaccine candidates for dengue are in various stages of clinical trials [2]. However, for most of the world’s population, vaccines for these viruses are currently either unavailable or too expensive. Clinical presentations can be ambiguous and diagnoses notoriously difficult based on symptoms alone. Laboratory confirmation is therefore often critical for diagnosis. While arboviral infections could potentially be treated using antivirals such as Ribavirin [3], and is occasionally treated with IVIG, currently the usual treatment is supportive therapy only. The presence of viral RNA in blood is typically fleeting, so antibody testing is often the method of choice to provide a laboratory diagnosis or to help rule in or rule out other more treatable infections.

A variety of techniques have been developed over the past 40 years for the serodiagnosis of arboviruses. These include immunofluorescence assay, complement fixation test, hemagglutination inhibition assay, plaque reduction neutralization test (PRNT) [4], and IgM and IgG enzyme-linked immunosorbent assays (ELISAs) [5,6]. The most recent addition to the menu of tests is the microsphere immunoassay (MIA) [7,8]. Currently, ELISAs and MIAs are generally used as screening tools to separate those specimens that are negative to the arboviral antibody tested for, from those that should receive confirmatory testing. In a known outbreak situation, IgM and IgG assays are sometimes performed without using confirmatory methods. A combined approach enables the broadest spectrum of information to be captured and interpreted in light of the clinical picture, any travel history of the patient, and timing of specimen collection.

A critical part of arboviral laboratory diagnosis pertains to the serologic testing for related viruses. Antibodies to one virus of a particular genus will frequently cross-react with heterologous antigens within the genus [7]. Much effort has been put into the development of tests and reagents that reduce or remove this cross-reactivity [9]. If successful, such methods would reduce the need for confirmatory testing with PRNT. However, the cross-reactivity seen using currently available reagents can be taken advantage of. The possession of an understanding of the cross-reactivities of these viruses both inform diagnoses, and help in the recognition of viruses formerly absent from a particular geographic region [10]. At the CDC Arboviral Diseases Branch, laboratory diagnosis typically employs the geographic approach to testing. The initial screening incorporates a number of arboviruses known to be present in the region of the world where the patient resides or has recently traveled to.

Microsphere-based immunoassays (MIAs) have been used as screening tools for arboviruses over the past 5 years. A number of US State and government labs including the CDC have used a duplex IgM tests for detection of antibodies to West Nile (WN) and St. Louis encephalitis (SLE) viruses [7], and have participated in proficiency testing using this method. This test was recently adapted for the detection of IgG to WN and SLE viruses (unpublished data). The speed and ease of use of these methods have made them attractive for expansion to other arboviruses, where viral antigens of interest can be incorporated into the testing battery. The body of work presented here elaborates on these methods and capitalizes on the multiplexing capability of the Luminex platform. The creation of IgM and IgG multiplex MIAs allows for a comprehensive array of arboviral infections to be tested for concurrently. The large and complex data set that results from extensive multiplexing necessitates a thorough investigation of classification methods in order to identify the most effective technique. It must allow both flexibility of use and the ability to accommodate the inherent cross-reactivity of these viruses. Here we report the development of multiplex microsphere immunoassays for detection of IgM and IgG to 6 flaviviruses, 6 alphaviruses, and 1 bunyavirus of human importance, incorporating validation results for its practical use in geographic batteries.

## Materials and Methods

### Ethics statement

The Division of Vector-Borne Diseases Human Subjects Advisor to the Centers for Disease Control and Prevention Institutional Review Board reviewed the procedures for “Multiplex Microsphere Immunoassays for the Detection of IgM and IgG to Arboviral Diseases” and confirmed that they do not meet the definition of research involving human subjects specified by 45 CFR 46.102(f). CDC IRB review was not required because specimens involved in this study were originally collected as part of standard CDC diagnostic operations and are archived expressly for development and testing. These specimens had all donor identification material removed at the time they entered the archive. Because data will be non-identifiable, this activity does not involve human subjects.

The suckling mouse brain antigens used in this study were made at the Centers for Disease Control and Prevention under the guidance of the Centers for Disease Control and Prevention-Fort Collins Institutional Animal Care and Use Committee (IACUC), protocol 11-013. Pain and suffering was minimized by hypothermia to effect during inoculation followed by return of the animals to their mother; euthanasia was performed at the first signs of illness including reduced milk intake. Animals were euthanized using isofluorane by inhalation to effect and hypothermia to effect as specified by the IACUC. Antigens produced under this protocol were not made specifically for this study but were made in accordance with the specific mission of the Centers for Disease Control and Prevention to provide reference quantities of reagents for arboviruses, and are made widely available.

### Specimens

Identifying information was removed from serum and cerebrospinal fluid (CSF) specimens obtained from the DVBD diagnostic archives (Tables 1 and 2). The samples had previously been screened for anti-arboviral antibodies using one of the currently employed diagnostic IgM protocols (specifically IgM-capture ELISA (MAC-ELISA) [5], WN/SLE IgM MIA [7], or EEE IgM MIA (unpublished method)) or one of the IgG protocols (IgG-ELISA [6] or WN/SLE IgG MIA (unpublished method)) for serum only. A confirmatory plaque reduction neutralization test (PRNT) [4] was previously performed for all serum samples that produced positive IgM screening results, and for all samples that were submitted specifically for vaccine titer confirmation. Panels of serum samples were assembled to determine the initial classification parameters for the multiplex IgM microsphere immunoassay (multiplex IgM-MIA) and multiplex IgG-MIA. Infecting viruses were: flaviviruses dengue (DEN), Japanese encephalitis (JE), Powassan (POW), SLE, WN, and yellow fever (YF); alphaviruses eastern equine encephalitis (EEE), Mayaro (MAY), Chikungunya (CHIK), Ross River (RR), Venezuelan equine encephalitis (VEE), and California serogroup bunyavirus La Crosse encephalitis (LAC). While alphavirus western equine encephalitis virus (WEE) antigen was included in the tests, samples were unavailable. A panel of samples previously resulting in negative arboviral diagnoses was assembled (NEG). Numbers of sera per virus are listed in Table 1 in the “Initial” serum columns. Additional samples were assembled to investigate the use of geographic batteries (Table 1 “Geo Val” columns) and finally, samples submitted to the DVBD Arbovirus Diseases Activity diagnostic lab in the summer of 2011 were analyzed to validate the methods (Table 1 “2011” serum columns). No samples were available for WEE virus, and the VEE and YF samples were from vaccinees. Additional panels of sera were compiled for syndromes clinically similar to some arboviruses as follows: syphilis (N=58), Lyme disease IgM (N=10), Lyme disease IgG (N=10), rheumatoid factor (N=13) and anti-nuclear antibody (N=22). To determine if the multiplex IgM method was useful for the testing of CSF a panel of 131 samples comprising IgM-positives to WN (N=24), LAC (N=11), and JE (N=3) and POW (N=3) viruses and YF vaccine (N=1), plus antibody-negative samples (N=89) was assembled.

**Table 1:** Samples tested in the IgM and IgG multiplex MIAs listed by virus.

	**IgM**						**IgG**				
	**Serum**			**CSF**			**Serum**				
Truth*	**Initial**	**Geo Val**	**2011**	**Initial**	**2011**	**Total**	**Initial**	**Geo Val**	**2011**	**Total**	
NEGIgM	79	64	222	19	70	454	93	54	173	320	
CHIKIgM	44	7	10	0	0	61	45	1	1	47	
DENIgM	64	22	3	0	0	89	72	0	1	73	
EEEIgM	34	0	2	0	0	36	39	0	0	39	
JEIgM	29	0	1	2	1	33	28	0	1	29	
LACIgM	33	4	41	5	6	89	35	0	9	44	
MAYIgM	4	1	2	0	0	7	4	0	1	5	
POWIgM	7	2	13	0	3	25	6	2	8	16	
SLEIgM	54	2	0	0	0	56	61	0	0	61	
VEEIgM	6	0	0	0	0	6	16	0	0	16	
WNIgM	66	3	24	12	12	117	78	3	23	104	
YFIgM	81	11	9	1	0	102	39	9	11	59	
Non-arbo	103	0	0	0	0	103	103	0	0	103	
**Total**	**604**	**116**	**327**	**39**	**92**	**1178**	619	69	228	916	

* Based upon previous IgM/IgG ELISA/MIA and PRNT results

**Table 2:** Samples tested by IgM and IgG multiplex MIAs listed by geographic battery.

	**IgM**						**IgG**			
	**Serum**			**CSF**			**Serum**			
**Battery**	Initial*	**Geo Val**	**2011**	Initial*	**2011**	**Total**	Initial*	**Geo Val**	**2011**	**Total**
**US**	273	42	233	36	80	**664**	312	31	175	518
**CSAM**	393	39	25	32	4	**493**	402	25	18	445
**AAE**	375	35	69	34	8	**521**	361	13	35	409

* Samples were tested in all batteries in which the infecting virus appeared

### Assay controls

Controls were developed to confirm that components had been added correctly to the tests and to confirm sample characteristics and system integrity. Flavivirus group-reactive MAb DEN 4G2 [11], alphavirus group-reactive MAb EEE 1A4B-6 [12], and California serogroup bunyavirus group-reactive MAb LAC 10G5.4 [13] were each conjugated to phycoerythrin by Prozyme Inc., San Leandro, CA. These were used to test for antigen reactivity by adding to a well containing the other test components. A negative serum sample, previously tested for antibodies to all arboviruses, was added to a well on the plate containing all the other test components to serve as a baseline for the assay. The following internal control sets of microspheres, made by Radix BioSolutions, Georgetown, TX, were added to all wells of the assay: control to monitor nonspecific binding of detection reagents, instrument reporter laser control, serum addition control (IgM + IgG), IgM reporter addition control (IgM test only), IgG reporter addition control (IgG test only) and rheumatoid factor control (RF, IgM test only). The internal controls were used with the summer 2011 evaluation samples only.

### Coupling of microspheres to monoclonal antibodies

Because purified antigens were not available for many of the viruses involved in the multiplex MIA, capture of the antigens was achieved using monoclonal antibodies (MAbs) coupled to the microspheres. Three MAbs were used: flavivirus group-reactive 6B6C-1 [14], alphavirus group-reactive 2A2C-3 [15], and anti-LAC 807-22 [13]. For each viral antigen, 25 µg of the appropriate purified antibody was covalently coupled to 5.4 million carboxylated MicroPlex® microspheres (Luminex Corporation, Austin, TX), using standard carbodiimide methodology. Hence, 6 microspheres sets of different spectral addresses were coupled to 6B6C-1 to accommodate the flaviviral antigens, 6 sets were coupled to 2A2C-3 (for the alphaviral antigens), and a single set was coupled to 807-22 (for the bunyaviral antigen). All coupled microsphere sets were adjusted to a concentration of 5x10^6^ microspheres/ml and stored at 4°C for up to 18 months thereafter, as determined empirically.

### Addition of antigens to coupled microspheres

Viral antigens were prepared in either suckling mouse brain (SLE, POW, YF, VEE, MAY, RR, CHIK, EEE, WEE, LAC) or were engineered recombinants expressed in COS-1 cells (WN [16], DEN 2 [17] combined with DEN 3 (unpublished), and JE [18]. Antigens were produced at the CDC/DVBD with the exception of the WN viral recombinant antigen, which was provided as a gift by Hennessy Research, Shawnee, KS. The optimal amount of viral antigen to add to the coupled microsphere sets varied depending on the individual antigen and varied between lots. This was initially determined by titration using known antibody-positive serum controls to each virus and compared to a negative serum control, and the working dilution was chosen to yield a median fluorescent intensity (MFI) of approximately 2000 for the positive controls. For the IgM assays, positive and negative sera were IgG-depleted using protein G sepharose prior to use. Antigen volumes per ml of antigen/coupled microsphere stock preparation ranged from 0.75 µl to 160 µl. In addition, negative (mock) antigens (suckling mouse brain (NSMB) and recombinant (NREC)) were added to separate sets of microspheres. The volumes used for the negatives were equivalent to those of the antigens for each virus family that required the most volume and were included in the test to identify nonspecific background reactions of the serum with both the coupling monoclonal antibody and any non-viral protein in the antigen preparations [19]. Coupled microspheres and antigens were mixed together in Candor Antibody Stabilizer (Boca Scientific, Boca Raton, FL) on a rotating platform for 1 h at room temperature, after which they were stored in the dark at 4°C for a minimum of 12 hours prior to use and up to 6 months thereafter, as determined by previous stability experiments (unpublished). Microspheres were at a concentration of 5 x 10^5^/ml in these stock solutions, which were generally made in 2 ml batches. The same preparation of antibody-coupled microspheres/antigen was used for both IgM and IgG MIAs for each virus. The microsphere regions (sets) used in these experiments were as follows: 6B6C-1 {11 (WN), 12 (SLE), 13 (POW), 14 (DEN2/3), 15 (JE), 16 (YF), 18 (NSMB), 19 (NREC)}; 2A2C-3 {21 (VEE), 22 (MAY), 24 (RR), 25 (CHIK), 26 (EEE), 27 (WEE), 28 (NSMB)}; 807-22 {51 (LAC), 52 (NSMB)}.

### Preparation of samples

For the IgM-MIA, it was desirable to remove potentially interfering IgG from the serum. The samples were diluted 1:20 in PBS then reacted with protein G sepharose in a 96-well filter plate Millipore Corporation, Billerica, MA) for 30 min at room temperature as previously described [7]. The plate was filtered using a vacuum manifold (Pall Corporation, Ann Arbor, MI) and the filtrates captured in a round-bottomed 96-well plate. Samples were further diluted to 1:400 [7] using 50% Low Cross Buffer in PBS pH 7.2 (Boca Scientific, Boca Raton, FL) prior to use. For the IgG-MIA, no prior treatment was necessary and samples were diluted to 1:400 in 50% LCB in PBS prior to use. To minimize pipetting errors and to make transfer to the MIA plates as convenient as possible, samples were placed identically on the serum dilution plates for IgM and IgG, in the order and position that they would appear on the MIA plates. Cerebrospinal fluid samples required no protein G treatment and were diluted 1:5 in 50% LCB in PBS.

### IgM and IgG-MIA

To maximize efficiency and conserve supplies, IgM and IgG-MIAs were prepared concurrently. Two filter plates were prewetted with 150 µl PBS, one for the IgM and one for the IgG assay. A cocktail of viral antigens/antibody-coupled microspheres was made that included all 13 regions of microspheres associated with the viral antigens. A volume of 5 µl per microsphere region for each assay well was added to a single polypropylene tube, and undiluted LCB was used to make up the total volume so that 150 ul/well of the cocktail could be added to both plates. Similarly a cocktail containing the negative antigens/antibody-coupled microspheres was made that included all 4 negative antigen sets, using 5 µl of each set for each well, and undiluted LCB was used to make up the volume so that for 50 µl/well could be added to both plates. The negative cocktail was vortexed thoroughly and divided into 2 equal parts: one for the IgM assay and one for the IgG assay. For the IgM test 0.25 µl/well each of the internal controls: nonspecific control (region 53), serum verification M+G control (region 30), instrument reporter laser control (region 97), rheumatoid factor (RF) control (region 42), and reporter control M (region 47) was added to the negative antigen cocktail. For the IgG test, 0.25 µl/well each of the internal controls: nonspecific control (region 53), sample control M+G (region 30), reporter laser control (region 97), and reporter control G (region 33) was added to the negative antigen cocktail. Antigen detection controls were prepared in 50% LCB in PBS (4G2-PE at 8 µg/ml; 1A4B-6-PE and 10G5.4-PE at 4 µg/ml). A negative control serum was diluted to 1:400 in 50% LCB in PBS. The PBS was suctioned from the plates using a vacuum manifold. During all vacuum and wash steps, care was taken so that the filters did not completely dry out, which can cause aggregation of the microspheres and inconsistent results. The viral antigen/antibody-coupled microsphere cocktail was vortexed, and 150 µl was added to all control and test wells on both plates. This was immediately suctioned through the plate and the wells washed twice with 150 µl of PBS. Fifty microliters per well of vortexed IgM negative antigen cocktail plus internal controls were added to the IgM-MIA plate, and similarly the IgG negative antigen cocktail plus internal controls were added to the IgG plate. The addition of the negative antigens as a separate step was performed in order to avoid any contamination of the negatives with unbound viral antigens that would occur if the cocktails of viral and negative antigens on their respective antibody/beadsets were prepared in one tube. The wells were washed twice with PBS using the vacuum manifold, and the undersides of the plates were blotted to prevent capillary leakage in the next steps. To the IgM plate, 50 µl per well of 4 µg/ml donkey anti-human IgM R-phycoerythrin (Jackson Immunoresearch, West Grove, PA) in 50% LCB in PBS was added. Fifty microliters of the antigen detection controls 4G2-PE, 1A4B-6-PE and 10G5.4-PE were added to the first 3 wells on the plate in that order, and 50 µl of the negative serum control was added to the 4^th^ well. The IgG-depleted test serum samples at 1:400 and the CSF samples at 1:5 were transferred from the preparation plate to the subsequent wells on the plate at a rate of 50 µl/well. To the IgG plate, the antigen detection and negative controls were added to the first 4 wells, and the test serum samples at 1:400 were transferred to the subsequent wells. The undersides of both plates were blotted and the wells were covered with plate sealer. The plates were vortexed for 10 seconds on a flat surface vortexer to mix the well contents, the undersides blotted, the plates covered with aluminum foil-lined lids, and placed on a rotary plate shaker. The IgM plate was shaken for 1.5 hours at room temperature. The IgG plate was shaken for 45 minutes at room temperature, washed twice with PBS, and 50 µl/well of donkey anti-human IgG R-phycoerythrin (Jackson Immunoresearch, West Grove, PA) in 50% LCB in PBS was added. The underside of the plate was blotted followed by vortexing to mix the contents of the wells. The plate was shaken a further 15 minutes then washed twice with PBS. The underside was again blotted and 100 µl/well of BioPlex sheath fluid (BioRad, Hercules, CA) was added. The contents of the wells were resuspended and the median fluorescent intensity (MFI) values were obtained for the individually identifiable microsphere sets corresponding to the different antigens in each well using a calibrated and validated BioPlex 100 machine (BioRad, Hercules, CA). During results acquisition for the IgG plate, the incubation step for the IgM-MIA was completed and wells were washed twice with PBS, the underside of the plate blotted, and 100 µl/well BioPlex sheath fluid was added. The plate was placed in the dark until the IgG-MIA results acquisition was finished, after which the contents of the IgM plate were resuspended, the plate blotted, and results acquired. This method was used to test the initial serum samples and CSF samples detailed in the specimens section (Table 1).

### Analysis methods

We implemented and evaluated 8 classification methods to select the approach that would provide the best performance over the range of data generated for the initial serum sample set. The methods considered were 1) simple classification by determining which antigen yielded the highest V/N (MFI of sample reacted on viral antigen /MFI of sample reacted on negative antigen) (MAX.V); 2) highest P/N (MFI of sample reacted on viral antigen /MFI of negative control reacted on viral antigen) (MAX.P); 3) individual antigen receiver operating characteristic (ROC) curve analysis; 4) linear discriminant analysis (LDA); 5) multinomial logistic regression (MLR); 6) support vector machines with linear basis (SVM-LIN); 7) support vector machines with radial basis (SVM-RAD); and 8) LogitBoost, a specific boosting method (LOGITBOOST) [20]. All methods were implemented in the R statistical software package (R Development Core Team (2012)) [21]. In order to apply the different classification schemes, each sample in the dataset was labeled according to its known infecting virus based on previous diagnostic results using IgM-ELISA, IgG-ELISA, and PRNT. The known negatives were so labeled. These labels constituted “truth” with respect to classification status. Models were developed for each classification method using truth versus the multiple, multivariate MFI measurements for the samples. Each classification method was fit to the full dataset and was then used to predict the infecting virus for each sample. A prediction error occurred when the model predicted a result other than “truth”. To provide a further measure of predictive performance, cross-validation was used, where the full dataset was divided in half randomly, ensuring that each virus set was equally split. One half of the data, the test set, was removed, and all the classification methods were fit on the remaining data – the training set. The resulting fits were then used to predict the infecting virus or negativity of the samples in the test set, and the resulting prediction was recorded and compared with the truth. This random split was repeated 10 times, each of which provided an estimate of the expected error rate for all the classification methods. Finally, these were averaged and recorded as cross-validation error estimates (and associated confidence intervals) for each of the methods. The method with the lowest cross-validation errors for both true positives (sensitivity) and true negatives (specificity) as well as for the overall error rate was chosen as the classification method for the multiplex MIA. If two methods were effectively tied in this performance, the method that was easier to interpret and to implement in an Excel Add-in (macro) for data manipulation was chosen.

### Validation based on geographic batteries

The IgM and the IgG-MIAs as described above contain 13 viral antigens. As a preliminary investigation into the use of geographic batteries, the panel of antigens in each multiplex was divided into 3 smaller panels: WN, SLE, POW, EEE, WEE and LAC for United States of America and Canada (US); WN, POW, DEN, JE, YF and CHIK for Asia/Africa/Europe (AAE); WN, SLE, DEN, YF, VEE, MAY, EEE and WEE for Central/South America (CSAM). The RR antigen was not included in any of the batteries. The data from the classifying sample set were allocated to all batteries containing the infecting virus. All 8 classification methods were applied, where the data used included only the information pertinent to the antigens in the assigned geographic battery. Separate classification rules were obtained for each analysis method for each geographic battery. An additional smaller sample set obtained from the DVBD diagnostic archives containing samples that were not included in the original serum set was assembled as a means to evaluate a modified laboratory methodology and to determine classification parameters specific to the geographic batteries (see Table 1 “Geo Val” columns for specimen details). The methods described above were modified to include only the specific geographic battery antigens in the antigen/bead cocktails. Hence, 3 viral antigen cocktails were prepared. Because the AAE and CSAM batteries did not include LAC, the bunyavirus control 10G5.4-PE was not included for these tests. The only other modifications for this set were that the reduced number of antigens allowed for the volumes of viral antigen cocktails per well to be reduced to 100 µl, and the wash volumes to be reduced similarly. For diagnostic purposes the usual setup would be to incorporate all 3 batteries on one IgM-MIA and one IgG-MIA plate in series, with the antigen detection and negative controls preceding each sample set for each battery. Results were analyzed based on the classification parameters created by splitting the initial sample set into geographic batteries.

### Summer 2011 samples used to validate the multiplex MIAs

Diagnostic serum and CSF submissions during the period May 2011 to September 2011 were tested for evidence of arboviral antibodies using current methodology to confirm positive reactions. After testing and reporting was complete, remaining samples were archived, de-identified, and used to validate the multiplex MIAs. A total of 419 samples were tested in the IgM multiplex MIAs; 228 samples were tested in the IgG multiplex MIAs (see Tables 1 and 2 for details). Results were analyzed using the most successful of the classification schemes (Logitboost and MLR) described above. 

## Results

The multiplex MIAs were initially performed to analyze serum and CSF antibodies reactive with 13 arboviral antigens, and to determine which classification method might be most successful for these data. The raw untransformed data showed that for many samples there was a discernible MFI difference between reactions with the homologous virus and the other arboviral antigens. Examples of these reactions for the IgM multiplex can be seen in Table 3. In the IgM test, approximately 91% of samples in the negative group had MFIs of less than 100 to viral antigens, whereas samples in the viral-positive groups had MFIs on a continuum from 100 to 10,000 to homologous viral antigens. The inherent background of the IgG test was higher than for the IgM test, with only 62% of negative samples exhibiting MFIs less than 100 to viral antigens, with the remainder varying up to 2,000. Samples in the viral-positive groups had MFIs of roughly 500 to 24,000. The overlap in MFI values between high negative and low positive samples, and between reactions to antigens of heterologous viruses are the scenarios in which choosing an appropriate classification scheme becomes particularly important. The overall variation and separation of the V/N and P/N measurements of samples tested on individual antigens in both IgM and IgG tests is illustrated in Figure S1a-d.

**Table 3:** Median fluorescent intensities (MFIs) on each antigen for selected samples tested in the IgM multiplex MIA.

	**Coupling Mab**															
	**6B6C-1**	**6B6C-1**	**6B6C-1**	**6B6C-1**	**6B6C-1**	**6B6C-1**	**6B6C-1**	**6B6C-1**	**2A2C-3**	**2A2C-3**	**2A2C-3**	**2A2C-3**	**2A2C-3**	**2A2C-3**	**807-22**	**807-22**
Dx result*	**WN**	**SLE**	**POW**	**DEN 2/3**	**JE**	**YF**	**NSMB**	N rec**	**CHIK**	**EEE**	**WEE**	**VEE**	**MAY**	**NSMB**	**LAC**	**NSMB**
**CHIK IgM+**	36	23	28	34	43	24	121	97	904	37	243	65	272	38	99	311
**DEN 1 1° IgM+**	672	256	184	7378	312	251	39	27	26	43	15	66	86	77	19	43
**DEN 3 2° IgM+**	41	47	20	2191	57	73	43	34	79	67	27	157	75	75	26	52
**EEE IgM+**	12	12	13	186	28	15	29	22	16	2915	164	99	220	20	36	62
**JE IgM+**	260	161	67	598	3850	75	159	135	74	74	27	246	493	97	184	341
**LAC IgM+**	6	6	10	46	5	13	21	15	14	20	9	50	77	43	1363	31
**MAY IgM+/YF+**	17	13	36	225	13	543	71	52	107	35	30	71	763	50	55	66
**POW IgM+**	7	15	5110	168	35	16	97	72	35	17	8	66	86	17	14	30
**SLE IgM+**	396	1950	47	1094	156	189	35	37	37	45	33	101	86	58	91	106
**VEE IgM+**	10	10	13	33	25	150	245	185	87	214	42	5072	136	216	98	98
**WN IgM+**	4908	284	35	455	221	101	62	35	44	50	26	195	183	76	97	126
**YF IgM+**	20	87	18	191	71	3990	16	13	10	16	9	26	58	25	21	32
**NEG**	14	14	36	70	26	31	135	119	10	20	10	37	139	32	21	43

* Diagnostic result

** Negative antigen

### Choice of classification method

To obtain an initial indication of which classification method would fit the multiplex MIA data the best, we surveyed 8 approaches that ranged from simple (such as highest V/N wins) to more rigorous methods that require complex computation. The methods evaluated were: 1) MAX.P; 2) MAX.V; 3) ROC; 4) LDA; 5) MLR; 6) SVM-LIN; 7) SVM-RAD; and 8) LOGITBOOST. Methods 3-8 used both V/N and P/N values, as it appeared that some viruses performed better with V/N than P/N and vice-versa, as illustrated in Figure S1a-d. Each measurement (V/N and P/N) was included so that their contributions to classification could be evaluated. Prediction error rates for the IgM multiplex MIA generated using these methods (except for ROC to which overall error rates do not pertain) showed that for the full dataset, the ranking of overall error rates were: LOGITBOOST < SVM-RAD < MLR < SVM-LIN < MAX.P < MAX.V < LDA. When cross-validation was applied to the data, overall error rates rankings were: LOGITBOOST < SVM-LIN < MAX.P < MAX.V < SVM-RAD < MLR < LDA. For the IgG multiplex MIA, the order of error rates were LOGITBOOST < MLR < SVM-RAD < SVM-LIN < LDA < MAX.P < MAX.V. Error rates from cross-validation of the IgG test were ranked: LOGITBOOST < SVM-LIN < LDA < SVM-RAD < MAX.P < MLR < MAX.V. Logitboost therefore yielded the lowest error rates for both assays for both the full data set and the cross-validation of the data sets. Using Logitboost, overall error rates of 0.4% and 0% resulted from the full data set for IgM and IgG assays respectively, and error rates of 11.5% and 8.3% resulted from the cross-validation for the IgM and IgG assays respectively. The higher error rates seen for cross-validation (“test”) are expected and provide a measure of anticipated prediction error for future use. Individual error rates for the viral antigens and overall rates for all the methods except ROC are shown in Tables 4 and 5. Based on this initial survey of methods where 13 viral antigens are included in the IgM and the IgG analyses, LOGITBOOST was the most successful of the classification methods that considers all virus groups simultaneously. It should be noted that to report sensitivities and specificities for these methods would be misleading because these metrics do not pertain to analyses of multiple viruses; hence error rates are a more informative and intuitive representation of truth.

**Table 4:** IgM multiplex MIA error rates (%) for each virus based on initial serum set.

			**LDA**		**SVM Linear**		**SVM Radial**		**Multi Lin Reg**		**Max. Value V/N**		**Max Value P/N**		**LogitBoost**	
**IgM**	**Virus**	**N**	Full*	Test**	**Full**	**Test**	**Full**	**Test**	**Full**	**Test**	**Full**	**Test**	**Full**	**Test**	**Full**	**Test**
	**CHIK**	46	30.4	29.4	6.5	8.7	0	47.8	0	34.8	15.2	12	4.4	4	0	15
	**DEN**	66	57.6	47.5	13.6	20.7	1.5	27.6	3	40.6	9.1	10.3	9.1	15.4	1.5	9.4
	**EEE**	38	52.6	50	10.5	19.1	2.6	57.1	0	42.9	15.8	5.6	21.1	11.1	0	5
	**JE**	29	17.2	0	6.9	10.5	0	21.1	0	64.3	6.9	9.1	3.5	0	0	30.8
	**LAC**	34	32.4	29.4	17.7	20	0	30	0	33.3	17.7	18.8	8.8	12.5	0	0
	**MAY**	4	25	100	0	0	0	0	0	100	50	100	75	100	0	66.7
	**NEG**	82	3.7	6.7	4.9	11.4	1.2	22.9	3.7	30.8	74.4	65.8	70.7	73.7	1.2	17.7
	**POW**	7	14.3	0	0	0	0	100	0	66.7	0	0	0	0	0	0
	**SLE**	57	28.1	42.9	8.8	7.7	0	11.5	0	16	10.5	13.8	3.5	3.5	0	4.4
	**VEE**	6	50	20	33.3	25	0	100	0	100	33.3	40	50	60	0	0
	**WN**	68	41.2	35.1	13.2	12.5	1.5	10	4.4	30.3	1.4	0	2.9	2.5	0	5.4
	**YF**	87	69	64.4	24.1	44.7	1.2	15.8	2.3	18.8	26.4	35.1	14.9	18.9	0	10.9
	**Overall**	524	38.4	36.4	12.4	19.2	0.9	28.6	1.9	35.1	23.3	21.4	19.9	20.3	0.4	11.5

* Full sample set was used to derive the classification parameters and error rates of entire sample set were determined based on these parameters.

** Test (cross-validation) pertains to the full sample set that was divided by 2 and one half was used to determine the classification parameters and the other half was tested using these parameters

**Table 5:** IgG multiplex MIA error rates (%) for each virus based on initial serum set.

			**LDA**		**SVM Linear**		**SVM Radial**		**Multi Lin Reg**		**Max. Value V/N**		**Max Value P/N**		**LogitBoost**	
**IgG**	**Virus**	**N**	Full*	Test**	**Full**	**Test**	**Full**	**Test**	**Full**	**Test**	**Full**	**Test**	**Full**	**Test**	**Full**	**Test**
	**CHIK**	45	17.8	9.1	4.8	7.8	3.8	11.8	0	19.2	77.8	63.2	20	21.1	0	0
	**DEN**	75	14.7	5.7	0	0	0	33.3	1.3	20	4	4.2	44	33.3	0	6.1
	**EEE**	40	15	10	5.3	22.5	4	20	0	40	35	33.3	15	9.5	0	12.5
	**JE**	28	35.7	30.8	2.5	5	2.5	15	0	23.1	14.3	5.9	7.1	0	0	9.1
	**LAC**	35	25.7	30.4	3.6	35.7	0	35.7	2.9	16.7	11.4	20	8.6	0	0	5.6
	**MAY**	4	0	0	5.7	10.5	2.9	31.6	0	100	0	0	100	100	0	0
	**NEG**	105	5.7	7.7	0	33.3	0	100	2.9	21.6	73.3	73.5	59.2	59.2	0	3.7
	**POW**	6	33.3	25	0	33.3	0	66.7	0	100	0	0	0	0	0	50
	**SLE**	62	29	18.2	3.2	13.8	1.6	24.1	0	24.1	19.4	17.2	0	0	0	18.8
	**VEE**	16	31.3	22.2	0	0	0	83.3	0	40	100	100	42.9	42.9	0	0
	**WN**	79	22.8	27.8	2.5	12.2	2.5	7.3	0	39	17.7	14	6	6	0	7.7
	**YF**	39	41	42.1	10.3	12.5	2.6	18.8	2.6	25	7.7	7.1	7.1	7.1	0	13.3
	**Overall**	534	20.4	18.2	3.9	12.6	2.4	22.6	1.1	28.4	33.9	29.6	22.6	22.6	0	8.3

* Full sample set was used to derive the classification parameters and error rates of entire sample set were determined based on these parameters.

** Test (cross-validation) pertains to the full sample set that was divided by 2 and one half was used to determine the classification parameters and the other half was tested using these parameters

Because ROC compares individual virus groups to the negative group only, it differs from the other classification methods, and error rates for this method are reported in Table 6. The ROC analysis included individual error rates calculated based on calculated cutoff values for V/N and P/N results, and also results using V/N and P/N values of 2 as a cutoff, in order to compare the calculated cutoffs to the traditional ELISA cutoff of 2 for all viruses. In the event that geographic batteries were not appropriate to use, ROC would be the method of choice. The decision to use V/N or P/N with a calculated ROC cutoff or a cutoff of 2 for the IgM test is virus-dependent, although V/N with a calculated ROC cutoff was generally more successful than the other variants. By contrast, clearly the best ROC method across all viruses in the IgG multiplex was to use V/N with a calculated cutoff. As might be anticipated, the instances where a cutoff of 2 improved the error rates compared to the data-derived cutoff, the calculated cutoff was close to 2. This indicated that as a general rule, cutoffs calculated for the individual viruses were more useful.

**Table 6:** Comparison of apparent error rates (%) generated by using calculated ROC cutoffs and cutoffs of 2.

	**IgM**					**IgG**				
	V/N		P/N			V/N		P/N		
**Virus**	Calc. cutoff	Cutoff=2	Calc. cutoff	Cutoff=2	N IgM	Calc. cutoff	Cutoff=2	Calc. cutoff	Cutoff=2	N Igg
CHIK	1.5* (1.1**)	2	4.5 (2.4)	4.5	61	0.0 (3.7)	0	1.7 (3.9)	10	47
DEN	5.6 (5.4)	20.7	4.4 (5.0)	7.8	89	4.6 (10.6)	24.9	14.9 (4.9)	23.2	73
EEE	2.6 (1.0)	**0.9**	6.7 (2.1)	6.7	36	0.3 (2.2)	0.6	1.8 (3.1)	4.7	39
JE	2.7 (2.9)	**2.4**	3.0 (5.9)	6.5	30	3.7 (2.6)	4.9	6.2 (9.5)	24.1	29
LAC	6.5 (1.9)	**6.2**	10.3 (3.0)	17	78	4.5 (2.9)	5.8	11.2 (4.2)	34	44
MAY	0.0 (14.5)	27.3	0.0 (6.7)	6.3	7	0.0 (9.1)	37.4	0.0 (4.2)	7.2	5
POW	0.3 (3.7)	5.8	0.9 (11.5)	10.4	22	0.6 (18.6)	6.9	1.9 (13.0)	28.9	16
RR	1.1 (5.0)	41.1	12.2 (1.3)	7.8	8	0.0 (6.3)	42.3	1.0 (3.1)	3.6	6
SLE	1.7 (4.1)	5.8	1.1 (11.2)	12.7	56	3.3 (3.8)	8.8	3.3 (13.0)	38.5	61
VEE	16.5 (1.8)	9.5	3.2 (1.9)	**3.2**	6	0.0 (4.2)	2.7	1.3 (2.8)	5.3	16
WN	0.9 (9.8)	5.7	2.2 (2.8)	2.8	93	1.8 (7.8)	9.8	3.0 (13.4)	16.3	104
YF	14.5 (2.4)	16	9.9 (2.1)	10.6	101	5.3 (5.9)	14.1	5.3 (11.3)	24.2	59
Total***	5	3	3+1 tied	1 tied		10+3 tied	0	3 tied	0	

* Lowest error rate per test shown in bold

** Calculated cutoff value

*** Total lowest error rate for each method of cutoff calculation

### Geographic batteries

**Figure 1:**
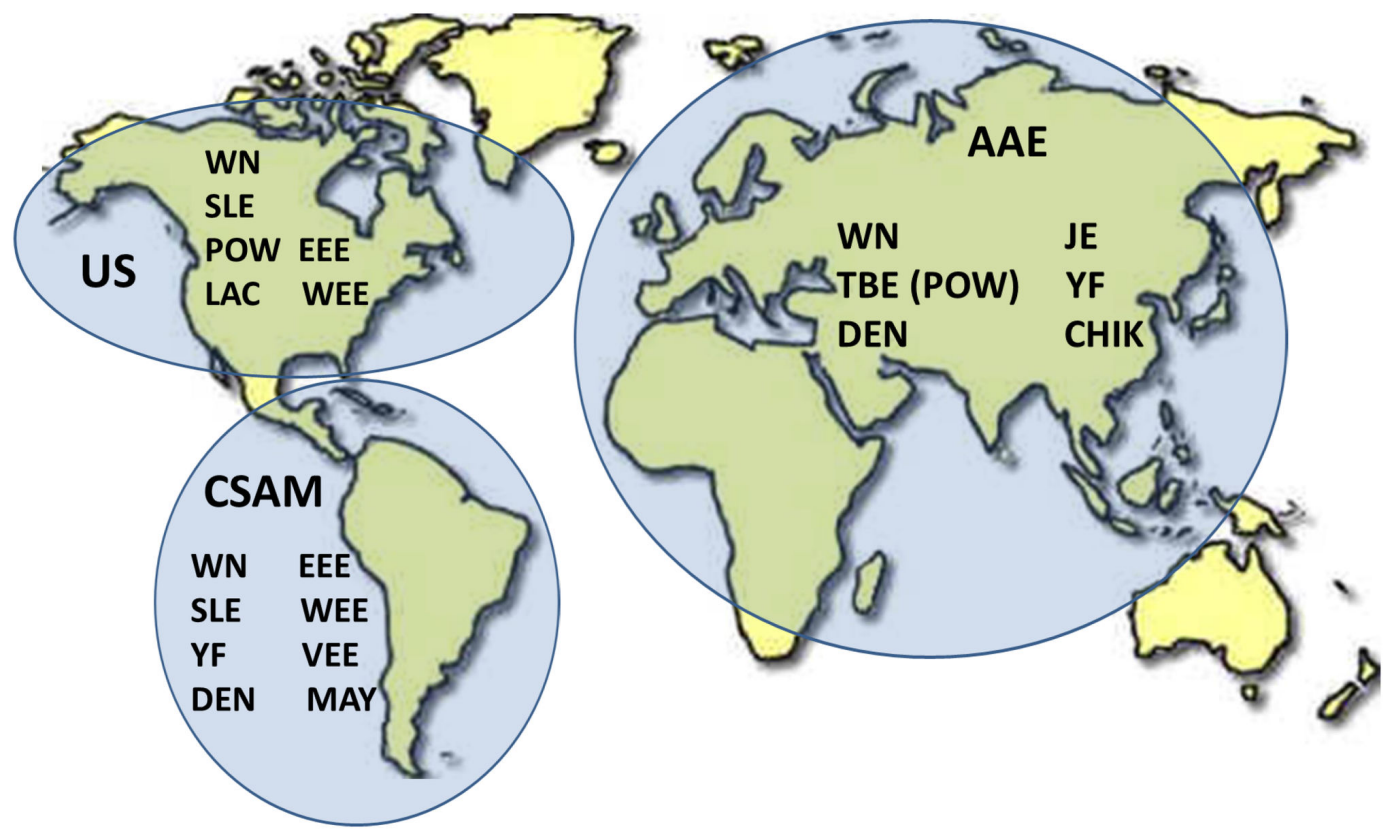
Map depicting the areas covered and viruses tested for in the 3 multiplex MIA geographic batteries. US covers the US and Canada; CSAM covers Central and South America and the Caribbean; AAE covers Asia, Europe and Africa. Australia and parts of the South Pacific are not included in the multiplex batteries.

Immunoglobulin M and IgG multiplex MIA data for each of the samples from the initial sample set (used to derive the classification parameters) were assigned to one of the 3 geographic batteries (US, AAE or CSAM). A map of the proposed batteries is shown in Figure 1. Only the data from the antigens within the batteries were used. New classification parameters (cutoffs or probabilities) were derived for each of the antigens within the batteries, and the error rates for each method using the full and test (cross-validation) sets are listed in Table 7 in rows labeled “Init (full/test).”. Error rates were calculated using both V/N and P/N values together. For the both the IgM and IgG analyses of the geographic batteries, LOGITBOOST gave the lowest error rates for both the full data set and cross-validations. Error rates for LOGITBOOST ranged from 0.4% (US)-1.9% (AAE) for the full set and 8.94% (AAE)-11.67% (CSAM) for the cross-validation in the IgM test; and from 1% (US)-1.2% (CSAM) for the full set and 7.9% (AAE)-11.6% (CSAM) for the cross-validation in the IgG test.

**Table 7:** Error rates (%) for all classification methods and geographic testing batteries.

**Method**	**Set**	**Type**			**IgM US**	**IgG-US**	**IgM CSAM**	**IgG CSAM**	**IgM AAE**	**IgG AAE**	**IgM All**	**IgG All**
**LDA**	**Init**	**Full**			30.1	18.0	40.7	18.8	41.3	24.7	38.2	20.6
	**Init**	**Test**			28.9	17.6	41.7	19.2	40.1	25.6	38.2	19.2
	**GeoVal**	**Eval**			11.1	0.0	22.5	16.0	21.6	20.0		
	**2011**	**Eval**			14.1	4.8	0.0	0.0	22.2	12.5		
**SVM - Linear**	**Init**	**Full**			10.5	2.8	14.2	6.0	15.3	9.0	12.4	3.9
	**Init**	**Test**			13.4	7.5	22.4	10.3	20.2	13.0	20.0	10.2
	**GeoVal**	**Eval**			2.2	3.1	7.5	4.0	10.8	13.3		
	**2011**	**Eval**			10.6	7.5	10.0	5.3	17.3	17.5		
**SVM - Radial**	**Init**	**Full**			3.5	1.2	2.0	2.1	4.4	2.9	1.7	1.5
	**Init**	**Test**			12.8	9.8	20.9	16.7	13.8	14.5	27.1	20.8
	**GeoVal**	**Eval**			24.4	9.4	32.5	12.0	18.9	20.0		
	**2011**	**Eval**			12.8	18.0	60.0	15.8	16.0	17.5		
**Multinomial**	**Init**	**Full**			1.8	0.6	5.9	3.1	6.2	3.7	1.9	1.1
	**Init**	**Test**			16.5	13.3	21.3	17.7	14.7	18.8	24.9	22.6
	**GeoVal**	**Eval**			26.7	31.3	22.5	76.0	16.2	53.3		
	**2011**	**Eval**			33.9	40.6	30.0	84.2	17.3	45.0		
	**Full**	**Full**			3.5	2.6	4.4	3.1	5.6	5.9		
	**Full**	**Test (95% CI**)			8.6 (7.5-9.6)	10.1 (8.9-11.2)	16.2 (14.7-17.7)	17.9 (16.3-19.5)	13.9 (12.5-15.2)	19.0 (17.4-20.7)		
**Logit Boost**	**Init**	**Full**			0.4	1.0	1.0	1.2	1.9	1.4	0.4	0.0
	**Init**	**Test**			6.3	7.7	12.3	8.1	9.6	11.2	12.4	12.3
	**GeoVal**	**Eval**			12.8	12.5	8.6	10.5	17.7	13.3		
	**2011**	**Eval**			21.3	14.8	30.4	12.5	12.1	12.9		
	**Full**	**Full**			1.3	1.0	1.1	0.7	1.9	1.7		
	**Full**	**Test (95% CI**)			7.2 (6.2-8.1)	5.9 (5.0-6.8)	9.7 (8.4-10.9)	9.7 (8.4-10.9)	7.2 (6.1-8.3)	9.0 (7.7-10.3)		
**MaxValue - V/N**	**Init**	**Full**			24.5	20.8	25.0	31.2	19.0	28.4	23.3	33.9
	**Init**	**Test**			23.8	20.8	16.3	32.2	19.8	29.1	21.2	33.7
	**GeoVa1**	**Eval**			64.4	75.0	27.5	24.0	46.0	20.0		
	**2011**	**Eval**			60.9	73.8	60.0	63.2	54.3	37.5		
**MaxValue - P/N**	**Init**	**Full**			20.6	20.2	21.6	27.6	17.1	27.1	19.9	28.3
	**Init**	**Test**			20.8	20.1	19.5	30.0	17.0	27.8	18.7	28.5
	**GeoVal**	**Eval**			57.8	87.5	47.5	68.0	27.0	46.7		
	**2011**	**Eval**			40.9	73.8	60.0	84.2	54.3	52.5		

A separate group of samples with roughly equal numbers spread between the geographic batteries were used to validate the classification parameters derived above (Table 1 columns “Geo Val”). Results in Table 7 (rows labeled “GeoVal”) show that the error rates are higher than those of the initial data set for most of the methods.

### Summer 2011 evaluation

**Figure 2:**
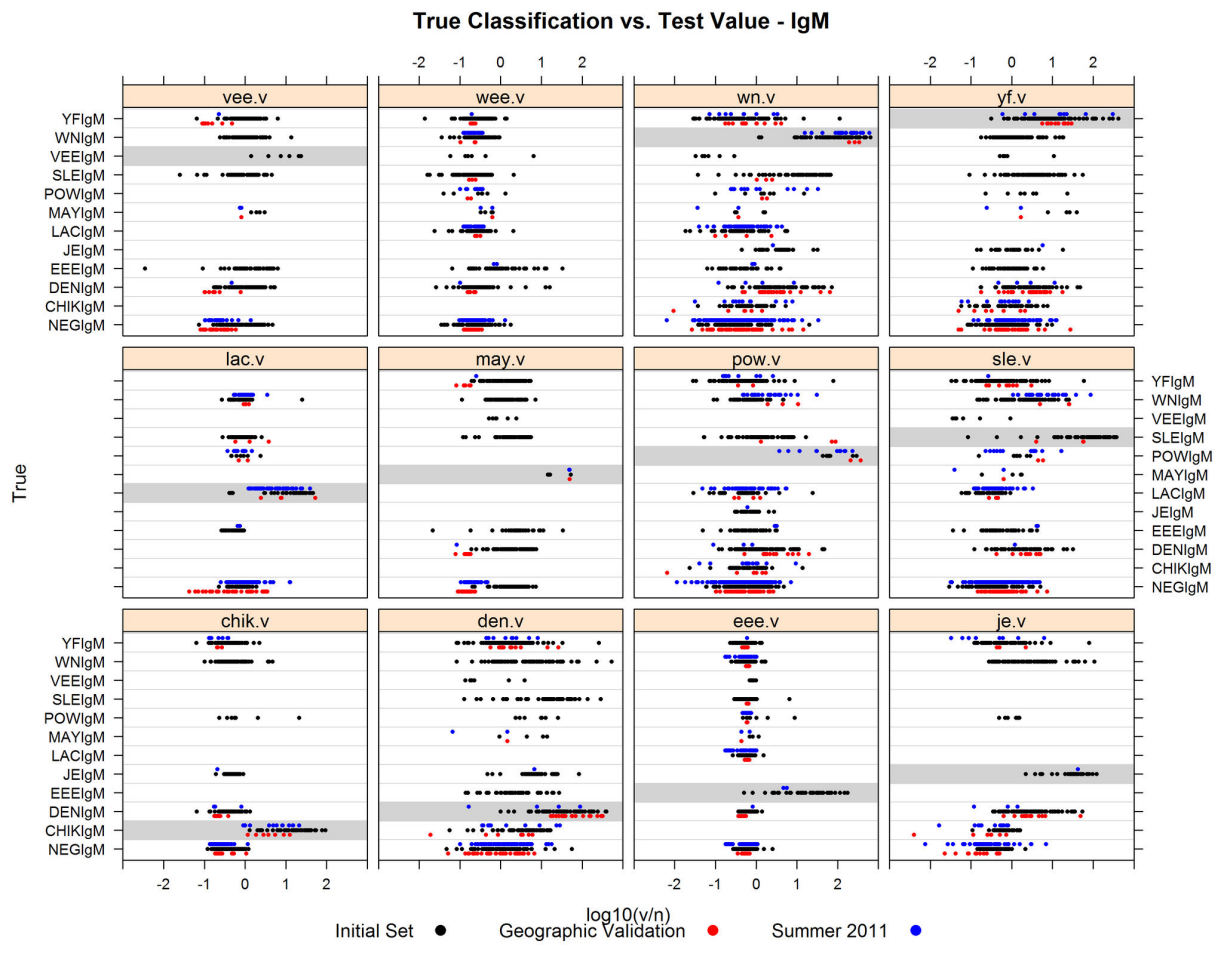
True classification vs. test value – IgM based on V/N. True classification (y-axis) refers to the original diagnostic result based on the traditional screening method plus plaque reduction neutralization, and test value (x-axis) is the V/N measurement for each sample. Samples in each set used in the analyses are depicted: black dots represent the initial set; red dots represent the geographic validation set; blue dots represent the summer 2011 set. Sample rows for the infecting virus are shaded grey in each antigen panel.

The classification parameters derived for the geographic batteries on the initial sample set were applied to IgM multiplex MIA results from 327 archived samples tested initially by the traditional methods over the summer of 2011, and to 228 IgG multiplex MIA results. The samples were divided into geographic groups based on their origins (Table 2). This dataset included samples that were equivocal, had dual infection, had unknown vaccination status, or were indeterminate. Analyses were performed on those samples that were diagnostically-conclusive using the 7 multivariate classification methods in order to generate error rates for the sample set (Table 7, rows “2011”). This served to identify any specific trouble spots. Multiplex MIA results from diagnostically indeterminate samples were reserved for later analysis (see the following section). Overall, LOGITBOOST gave the lowest error rates, but these rates were notably high for some of the viruses within the batteries, in particular LAC in the US IgM battery, and the negative groups in the US and CSAM batteries. The source of these errors is illustrated in Figure 2, where the V/N values of each sample in the original set, the geographic validation set, and the summer 2011 set for the IgM test are plotted for each virus. Using Lac as an example, the V/N’s of the original and geographic validation samples are higher overall than for the summer 2011 set. This caused several false negatives to be generated. False positives to SLE and EEE were also observed.

### Combination of samples to form the final models

**Figure 3:**
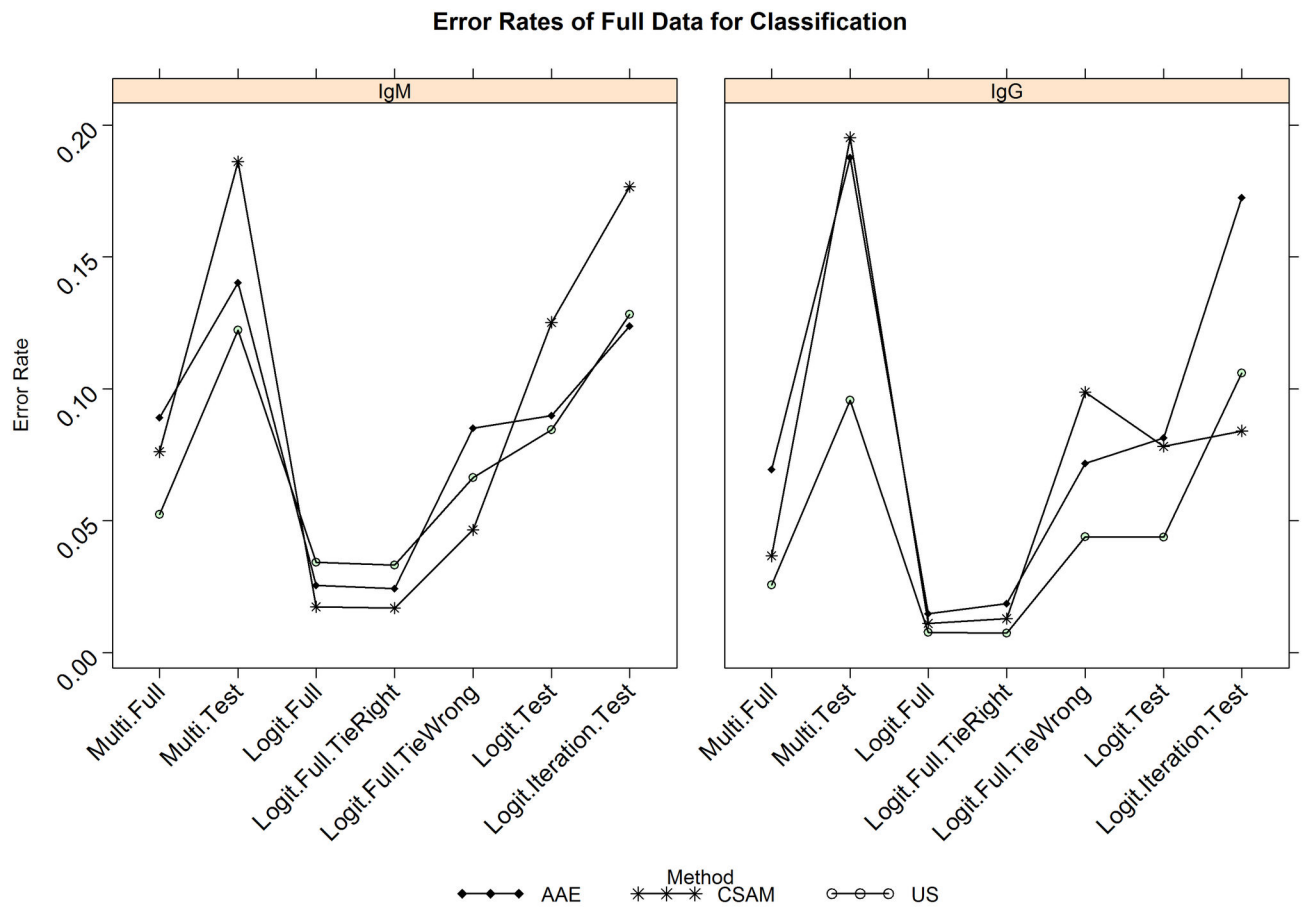
Error rates (%) for full data classification. Error rates (y-axes) where 1.0 is 100% incorrect classification (i.e. includes false positives and false negatives) are shown for the combined data set comprising the initial, geographic and summer 2011 sample sets. Error rates are shown for the 2 best classification options, multinomial linear regression (Multi) and logitboost (logit) on the x-axes. Full and test error rates are described in the analysis section of Materials and Methods. Logit. tie right/tie wrong is where the results that are tied are included in the error rates as being right or wrong. Logit. iteration is where more iterations of the classification scheme are performed than normally would be (usually equal to the number of data points in the set). This results in increased error rates due to over-fitting of the data such that any sample variation outside of the limits of the sample set used for deriving the classification rules results in an error.

To address the errors illustrated in Figure 2, all 3 datasets were combined, effectively doubling the number of data points. In addition, the amassed data were reviewed manually to identify any samples that yielded obviously spurious results, to avoid creating final classification parameters that would be unduly biased resulting in unnecessary error. These samples were removed from the analysis. The models were refitted on the resulting larger sets. Error rates for the geographic batteries using LOGITBOOST and MLR were improved considerably (Table 7 rows “Full set”), and are depicted in Figure 3. Error rates are also shown to illustrate the negative consequences of over-fitting the data using LOGITBOOST (“Iterations”), to confirm that the number of fitting iterations used in the final model was near-optimal. Because LOGITBOOST can result in a tie, error rates are also shown for when ties predict the correct (tie right) and incorrect (tie wrong) infecting virus. Indeterminate samples from 2011 (equivocals and dual infections) were analyzed using LOGITBOOST and MLR and results are illustrated for IgM only (Table S1). The applications of post-processing methods are needed to identify these types of samples during clinical use of the multiplex MIAs. Overall, LOGITBOOST (where both V/N and P/N values were included in the model as separate measures) was more successful than MLR, and was therefore selected as the classification method of choice.

### Cerebrospinal fluid

Thirty seven CSF samples were tested in the 13-virus IgM multiplex MIA format and analyzed using LOGITBOOST and MLR in the geographic batteries in which the infecting virus appeared, less two that were diagnostically indeterminate. In addition, 71 (US), 7 (AAE) and 3 (CSAM) CSF’s that were part of the summer 2011 validation sample set were analyzed using LOGITBOOST and MLR, with 12 indeterminate samples omitted. The infecting virus was identified correctly for 91% (US), 94% (CSAM) and 83% (AAE) of the samples using MLR. The correct virus identification was made for 90 (tie wrong)-92% (tie right) (US), 89-97% (CSAM) and 86-95% (AAE) of samples using LOGITBOOST. Details are shown in Table 8.

**Table 8:** Multinomial and Logitboost outcomes for CSF.

**US battery**							**CSAM battery**						**AAE battery**					
**Multinomial**							**Multinomial**						**Multinomial**					
	**Truth**							**Truth**						**Truth**				
**Predicted**	**LAC**	**NEG**	**POW**	**WN**	**Pred Total**		**Predicted**	**NEG**	**WN**	**yf vacc**	**Pred Total**		**Predicted**	**JE**	**NEG**	**WN**	**yf vacc**	**Pred Total**
**LACIgM**	**8**				8		**NEGIgm**	**21**	1		22		**JEIgM**	**2**		3		5
**NEGIgM**	2	**74**		1	77		**WNIgM**		**11**		11		**NEGIgm**		**24**	1		25
**POWIgM**		1	**3**		4		**YFIgM**	1		**1**	2		**POWIgM**	1	1			2
**SLEIgM**				2	2		**Truth total**	22	12	1	35		**WNIgM**			**8**		8
**WNIgM**	1	1		**21**	23								**YFIgM**		1		**1**	2
**Truth total**	11	76	3	24	114								**Total**	3	26	12	1	42
**US battery**							**CSAM battery**						**AAE battery**					
**LogitBoost**							**LogitBoost**						**Logitboost**					
	**Truth**							**Truth**						**Truth**				
**Predicted**	**LAC**	**NEG**	**POW**	**WN**	**Pred Total**		**Predicted**	**NEG**	**WN**	**yf vacc**	**Pred Total**		**Predicted**	**JE**	**NEG**	**WN**	**yf vacc**	**Pred Total**
**LACIgM**	**6**		1		7		**NEGIgM**	**20**	1		21		**JEIgM**	**3**		1		4
**NEGIgM**	2	**74**		1	77		**WNIgM**		**10**		10		**NEGIgM**		**23**	1		24
**POWIgM**		1	**1**		2		**YFIgM**			1	1		**WNIgM**			**9**		9
**WNIgM**	1	1		**23**	25		**Tie**	2	1		3		**YFIgM**				**1**	1
**Tie**	2		1		3		**Truth total**	22	12	1	35		**Tie**		3	1		4
**Truth total**	11	76	3	24	114								**Total**	3	26	12	1	42

### Non-arbovirus serum samples

Serum samples known to be positive for syphilis, Lyme disease IgM, Lyme disease IgG, rheumatoid factor and anti-nuclear antibody were tested using the multiplex IgM and IgG MIAs using the entire 13-virus panel to look for any cross-reactivity. No samples showed evidence of reactivity with any of the viral antigens in either of the multiplexes.

### Assay controls

Assay controls were added to the IgM and IgG MIAs for each geographic battery. A negative serum control served as the denominator for P/N calculations. The negative serum controls from the initial sample plates were compared to determine the variability between plates (intra-class correlation (ICC)) and whether there would be a need for subsequent test plates to be standardized against the historical controls. We evaluated the V/N values for the negative controls on all the viral antigens for within plate and among plate consistency by computing ICCs and associated 95% CIs. ICC values ranged from 0.87-0.99 with the confidence limits varying from 0.67 (WN) to 0.97 (LAC).

Genus-specific monoclonal antibodies coupled to phycoerythrin served as antigen verification controls. Means and standard deviations (SDs) were calculated for these controls on the viral antigens of their genus, and acceptable ranges were established (data not shown). These ranges will form the basis for quality control of subsequent diagnostic assays, once the method is introduced into the laboratory on a routine basis, and will be included in the assay analysis software that is currently under construction. Means, SD’s and 95% content and 95% upper and lower tolerance limits of the MFIs were calculated for internal control bead sets that were placed in each test well. Any sample MFI that fell below the lower 95% tolerance limit for one or more of the instrument reporter laser, serum or conjugate controls would be repeated. Sample MFIs for the nonspecific bead reaction control that were greater than the 95% confidence limit for that bead region would also be repeated. MFI values for RF were informational only (See Table S2 for details). Of the 419 serum and CSF samples tested for IgM in 2011, numbers of samples out of range were 2 (serum), 12 (RF), 2 (reporter control) and 2 (nonspecific bead reaction). Of the 228 serum samples tested for IgG in 2011, numbers of samples out of range were 1 (serum), 7 (reporter control) and 0 (nonspecific bead reaction).

### Dengue and yellow fever

The antigens used for DEN were a combination of recombinant DEN 2/3 serotypes; YF was 17D (vaccine strain) antigen made in suckling mouse brain. The individual performances of the known positive dengue and yellow fever (vaccine) serum samples in the13-virus multiplex MIA tests were assessed to determine a) whether the dengue antigen combination is sufficient to detect all serotypes from both primary and secondary infections, and b) to assess whether yellow fever vaccine recipients could be misclassified as being infected with alternate flaviviruses. Results from dengue serum samples of all 4 serotypes comprising both primary and secondary infections were analyzed using LOGITBOOST. Correct classification was achieved for 98% and 99% of results from the IgM and the IgG multiplexes, respectively, indicating that all 4 serotypes, regardless of whether they are primary or secondary, are capable of being classified correctly using LOGITBOOST. The single false classification for each test resulted from a secondary infection (IgM) and a primary infection (IgG). In addition, 91% of IgM samples gave classification probabilities of >90% for DEN. In the IgG test, 89% of samples gave classification probabilities >90% for DEN. Thus, the likelihood of a DEN infection being misclassified as another flavivirus was shown to be minimal using this analysis method. Using ROC (the individual method of analysis) 94% and 97% DEN-positive sera had values of V/N and P/N respectively that were greater than the calculated ROC cutoffs (Table 6) in the IgM assay. In the IgG assay, 95% and 84% had V/N and P/N values respectively that were greater than the DEN ROC cutoffs. In the IgM test, 1 primary and 4 secondary samples had V/N values below the cutoff; 1 primary and 1 secondary were below the P/N cut off. In the IgG test, 4 primary samples gave V/N values below the cutoff; in the IgG test 9 primary and 0 secondary samples were below the P/N cutoff. To obtain a measure of cross-reactivity between the flaviviruses as measured by ROC analysis, V/N values for the known DEN-positive samples tested on heterologous flavivirus antigens were analyzed. Values were found to be greater than the ROC cutoffs for WN (68%), SLE (35%), POW (2%), JE (60%), and YF (60%). When the absolute V/N values were compared, DEN V/N’s were 7 to 18 times higher to the dengue antigen than to the other viruses. The same trend was true for the IgG test, where 100% of DEN V/N’s were 2.5 to 5 times greater to the dengue antigen than to the other viruses. To test whether YF vaccine might be erroneously classified as a different flaviviral infection using the IgM test, the same type of V/N comparison was performed for the samples from YF vaccine recipients. Positive ROC results were obtained to WN (7%), SLE (7%), POW (1%), DEN (14%) and JE (7%), where the YF V/N’s were 5.5 to 19-times greater to the YF antigen than to the other viruses.

### Cross-reactivity of flaviviruses in the multiplex MIA versus ELISA

To partially evaluate whether the multiplex MIA demonstrated flavivirus cross-reactivity greater or less than the standard screening ELISA, an Arbovirus Diseases Branch database search was performed for IgM and IgG ELISA results of sera from confirmed SLE cases, because SLE represents one of the most serologically cross-reactive viruses in the genus. Results were compared from SLE antibody-positive samples where antibodies to DEN and WN viruses were also tested for, and where any samples with P/Ns of >2 were considered positive. The cross-reactivity’s seen for IgM ELISA using SLE-IgM positive samples were: DEN 42% (N=54); WN 85% (N=108). The cross-reactivity’s seen for IgG ELISA using SLE-IgG positive samples were: DEN 31% (N=26); WN 85% (N=64). By comparison, the multiplex MIA results using ROC cutoff’s shown in Table 6 gave cross-reactivity’s using SLE-IgM positive samples of: DEN 78% (N=54); WN 68% (N=54). The cross-reactivity’s seen for multiplex MIA IgG using ROC cutoff’s with SLE-IgG positive samples were: DEN 86% (N=59); WN 78% (N=59). LogitBoost results for the IgM multiplex MIA where the probabilities were >20% of the 59 SLE-positive samples being classified as DEN and WN were 13% and 0% respectively and where the probabilities were >10% of being classified as DEN and WN were 20% and 0% respectively. For the IgG multiplex MIA, the probabilities at the 20% Logitboost level were 2% and 2% for DEN and WN respectively; at the 10% level, probabilities were 17% and 5% respectively.

## Discussion

The multiplexing capability of the BioPlex (Luminex) platform allows for a single small sample to be simultaneously tested against multiple viral antigens, which is advantageous over methods such as ELISA because results are generated at the same time under the same conditions. The ability of these assays to incorporate internal controls further validates the results. From a practical standpoint, the ability to prepare reagents for several months of testing at one time streamlines the routine use of the multiplex MIAs. To facilitate the practical setup of these multiplex tests, an Excel® workbook was devised to calculate the amounts of reagents needed per test based on sample origin, to guide sample/plate orientation, to track lot numbers of reagents and to provide specific operating procedures. The multiplexes also reduce buffer usage and plastics consumption.

The challenge when dealing with the large amount of data produced by these assays is to devise a method that successfully harnesses the power of the multiplexing arrangement, produces an accurate result output, and is programmable for everyday use. Quadratic discriminant analysis, used previously [7], was unsuitable in the context of these expanded multiplexes. Therefore we compared 8 different analysis methods in IgM and IgG multiplexes, and evaluated the best of these by using additional samples assigned to geographic batteries based on the sample origin. The method that emerged as most useful was LogitBoost. For practical purposes, the probabilities generated by this method can be used not only to indicate the infecting virus, but also to rank close contenders that might have probabilities greater than for being negative. This informs the decision of which cross-neutralization tests should be performed, if applicable. LogitBoost has the advantage of potentially producing tied results, useful for identification of dual infections or equivocals. The resulting algorithm is relatively easy to program in Visual Basic® in Excel® (Microsoft Corp., Redmond, WA). The comprehensive output generated for the multiplexing method by LogitBoost captures and presents the data in a way that individual antigen analysis methods cannot accomplish, and the comparative error rates for the analysis methods underscore this utility.

The serodiagnostic portion of the clinical case definition adopted by the CDC for these viruses takes the following general format: Fourfold or greater change in virus-specific serum antibody titer (in quantitative tests between acute and convalescent specimens), or virus-specific IgM in cerebrospinal fluid (CSF), or virus-specific IgM demonstrated in serum and confirmed by demonstration of virus-specific IgG in the same or a later specimen by a different type of serological assay. A case will be classified as probable if confirmatory test results are not obtained [22]. Results from IgG testing using ELISA or MIA do not factor into the clinical case definitions for these viruses. However, the data from IgG tests are useful in a) corroborating results of the IgM tests, b) providing evidence of a previous arboviral infection when IgM is not detectable, c) indicating an anamnestic response, especially in secondary DEN viral infections, and d) pointing the diagnosis toward an unsuspected arboviral infection due to the presence of intra-genus cross-reactive IgG antibodies.

For the past 15 years, the Arboviral Diseases Branch at CDC has used ELISA to test for IgM and IgG to arboviruses [5,6]. Testing involves the reaction of an aliquot of diluted serum on a separate plate for each arbovirus indicated by the domestic or travel history of the patient, which may amount to 6 or more plates. This can be an inefficient use of time, reagents, sample and supplies. Recently, MIAs have been developed to detect IgM antibodies to WN and SLE viruses in a duplex arrangement [7], and also to detect IgM to EEE virus (unpublished). To perform the IgM and IgG multiplex MIAs in their entirety for each sample on a routine basis would be excessive and wasteful of reagents. The method used for triaging samples in the Arbovirus Diseases Branch diagnostic lab at the CDC is to test them according to a battery of arboviruses known to circulate in particular geographic locations. Currently, there are 9 geographic batteries (western US, eastern US, Europe, Asia, Central America, Africa, South America, South Pacific and Australia). To strike a balance between efficiency in use of reagents and complexity of test setup, the number of batteries for the MIAs was reduced to 3 (US, Asia/Africa/Europe (AAE), and Central/South America (CSAM), which took advantage of the large degree of duplication of viruses within these expanded batteries. Australia was not included as the only antigen in the multiplex specific to Australia is RR, and this can be performed *ad hoc*. An antigen preparation suitable for detecting anti-Murray Valley encephalitis virus antibodies using MIA was not available, so this and some other rarely needed tests will remain as ELISAs for the time being in our laboratory. The new geographic battery virus allocation appeared to work well and will simplify workflow.

The increased error rates seen when the geographic validation sets were tested is largely due to the relatively small numbers of samples for each virus within the groups; therefore one wrong result can make a large difference in error rate. As with any statistically-based model, the more data points there are in a set, the more accurate the predictions. To achieve this, all 3 data sets were combined to produce a final working model for use in the laboratory. This improved the error rates considerably for some groups. It should be noted that some viruses such as MAY were poorly represented and the strength of the models for these were not as great. In situations where only a few known positives with high V/N and P/N’s were available to establish the classification rules, there is the possibility that true positives with much lower values could occur. These may be classified incorrectly as negative, as the cutoff, regardless of classification method, would be impossible to determine accurately. To address this issue and those of background reactions and equivocal results, post-processing of results will be implemented within the context of an Excel® add-in which is currently in development. This will integrate with the Excel output of the BioPlex instrument to generate probabilities by using LOGITBOOST. Results for a specimen reacted on each viral antigen in the test batteries will be ranked, where the antigen with the greatest classification probability is reported as the infecting virus. The ROC data reported here gives individual V/N and P/N cutoffs for each viral antigen, and these can be used as a secondary measure to cross-check the results. For viruses where ROC cutoffs were derived from very small numbers of samples (e.g., MAY, POW, WEE) V/N and P/N cutoffs of 2.0 may be used, as confidence was low in the empirically-derived cutoffs and 2.0 is a number that has traditionally been used with ELISA, despite the low error rates seen with the calculated cutoffs. Additional post-processing will be used to identify the following categories: a) background reactions due to nonspecific activity of the samples to antigens causing false positive results, where V/N < ROC cutoff and P/N > ROC cutoff; b) indeterminate results where V/N > ROC cutoff but P/N < ROC cutoff; c) V/N and P/N are both > ROC cutoff, but LOGITBOOST probabilities are too close to call such that the infecting virus cannot be identified; d) equivocal results where the highest probability is close to that of the negative group. It should be noted that the outputs of the multiplexed arboviral MIAs are not quantitative in terms of comparing the amount of specific antibody in a sample.

The reasonably common situation arises where a sample needs testing for a virus that does not appear in the geographic battery related to its origin or is newly recognized as being important, for example Jamestown Canyon in the US and Canada [23] or recently, where DEN has been shown to be transmitted in the Florida Keys [24]. In this type of situation, viral antigens can easily be tested for on an *ad hoc* basis in addition to any geographic battery, and ROC can be used to determine reactivity to these antigens outside of the Excel add-in. Arboviral serosurveys that usually involve the testing of samples with only one or two antigens can utilize ROC cutoffs alone. However, LogitBoosting can conceivably be used under these circumstances with some further programming for use with flexible batteries, an ideal we are pursuing.

The ICC data suggested a small degree of plate to plate variation but the decision to normalize plates to mitigate this effect will be made when the Excel® Add-in had been completed, so that results can be compared for some positive samples using the finalized algorithm. It was observed that when the magnitude of the MFIs of the negative controls on a plate varied, the test samples varied similarly; hence the need for plate to plate comparison may be mitigated.

This study contains data regarding previously-understood but unpublished information regarding the degree of cross-reactivity between arboviruses, which is useful for purposes of test development and interpretation of results. This was discussed briefly in relation to DEN and YF. In addition, a glimpse into the cross-reactivity of ELISA versus multiplex MIA was illustrated by looking at the results when SLE-positive samples were tested in WN and DEN assays. The ROC cutoff method for MIA showed that cross-reactivity is detected in the multiplex as much if not more than in the ELISA (possibly due to a marginally greater sensitivity of the MIA), but that LogitBoost can be expected to yield greater viral specificity when applied to the MIA. In addition, these data may provide insight regarding the capability of combined IgM and IgG testing results to reduce the need for confirmatory PRNT’s in some instances. An in-depth analysis of both of these facets, while of great interest, is outside the scope of the current paper.

The Luminex platform has been used extensively for multiplexed testing for viruses in the diagnostic arena. The vast majority of these tests are for identification of virus-specific nucleic acid material [25,26]. Recently, multiplexing methods for serodiagnosis have been developed [27,28]. Many of these, such as Lammie et al. 2012 [29], take advantage of the method to test for etiologic agents of a disparate nature where antibodies are easily identified from one another. The choice of classification method may not be particularly critical under these circumstances. Here, we focus on very closely-related arboviruses, the antibodies to which frequently cross-react among the genuses, thus rendering analysis more challenging. This work represents both the most comprehensive, validated multiplexing method for arboviruses to date, and also the most systematic attempt at determining the most useful classification method for use with these serologic tests.

## Supporting Information

Table S1 **Multiplex results for summer 2011 samples with indeterminate or unconfirmed diagnostic IgM results.**
(XLSX)

Table S2 **Internal control data based on summer 2011 samples.**
(XLSX)

Figure S1 **True classification versus test values of initial data set: Figure S1a) V/N of IgM results; Figure S1b) P/N of IgM results; Figure S1c) V/N of IgG results; Figure S1d) P/N of IgG results.**
(TIF)
