# Supplementary figures and images for: Multiplex Microsphere Immunoassays for the Detection of IgM and IgG to Arboviral Diseases
Source: PLoS One. 2013 Sep 25;8(9):e75670. doi: 10.1371/journal.pone.0075670 (PMC3783417; doi:10.1371/journal.pone.0075670)

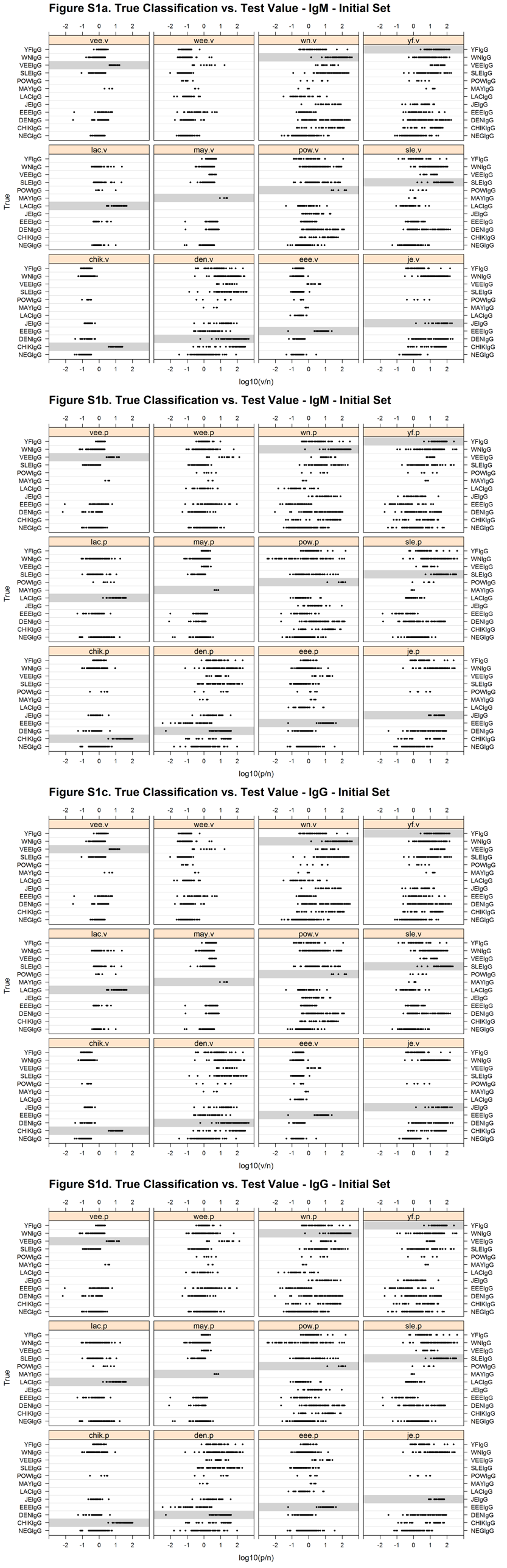

Supplement: Figure S1 — True classification versus test values of initial data set: Figure S1a) V/N of IgM results; Figure S1b) P/N of IgM results; Figure S1c) V/N of IgG results; Figure S1d) P/N of IgG results. (TIF) [file pone.0075670.s003.tif]
